# Associations between cadmium exposure and whole-body aging: mediation analysis in the NHANES

**DOI:** 10.1186/s12889-023-16643-2

**Published:** 2023-08-31

**Authors:** Ya Zhang, Mingjiang Liu, Ruijie Xie

**Affiliations:** 1 Department of Gland Surgery, The Affiliated Nanhua Hospital, Hengyang Medical school, University of South China, Hengyang, 421002 China; 2 Department of Hand & Microsurgery, The Affiliated Nanhua Hospital, Hengyang Medical school, University of South China, No.336 Dongfeng South Road, Zhuhui District, Hunan Province Hengyang, 421002 China; 3 Hengyang Medical school, University of South China, Hengyang, 421002 China

**Keywords:** Cadmium exposure, Biological aging, NHANES, Phenotypic age, United States

## Abstract

**Introduction:**

Even though cadmium (Cd) exposure and cellular senescence (telomere length) have been linked in previous studies, composite molecular aging biomarkers are more significant and reliable factors to consider when examining the connection between metal exposure and health outcomes. The purpose of this research was to assess the association between urinary cadmium (U-Cd) and whole-body aging (phenotypic age).

**Methods:**

Phenotypic age was calculated from chronological age and 9 molecular biomarkers. Multivariate linear regression models, subgroup analysis, and smoothing curve fitting were used to explore the linear and nonlinear relationship between U-Cd and phenotypic age. Mediation analysis was performed to explore the mediating effect of U-Cd on the association between smoking and phenotypic age.

**Results:**

This study included 10,083 participants with a mean chronological age and a mean phenotypic age of 42.24 years and 42.34 years, respectively. In the fully adjusted model, there was a positive relationship between U-Cd and phenotypic age [2.13 years per 1 ng/g U-Cd, (1.67, 2.58)]. This association differed by sex, age, and smoking subgroups (P for interaction < 0.05). U-Cd mediated a positive association between serum cotinine and phenotypic age, mediating a proportion of 23.2%.

**Conclusions:**

Our results suggest that high levels of Cd exposure are associated with whole-body aging.

**Supplementary Information:**

The online version contains supplementary material available at 10.1186/s12889-023-16643-2.

## Introduction

Cadmium (Cd) is a toxic heavy metal that is ubiquitous in the environment and poses a major public health challenge [1, 2]. The major sources of Cd exposure for the general population are tobacco smoke, diet, and workplace exposure [3–5]. Excessive exposure to Cd may lead to renal tubular dysfunction and abnormal bone metabolism [6–8]. In addition, there is evidence that Cd exposure induces oxidative stress, leading to elevated levels of inflammation and mitochondrial damage [9, 10].

Population aging is a global issue, with one-fifth of the world’s population expected to be 65 or older by 2030. Healthy life expectancy, on the other hand, is growing more slowly than total life expectancy [11, 12]. Although everyone ages, the pace at which biological aging occurs varies, and inequalities in aging rates between individuals manifest as differences in mortality and disease vulnerability [13]. Several aging metrics based on molecular factors, such as DNA methylation age, have been proposed [14], pro-inflammatory cytokines [15], and telomere length [16]. In addition, ‘phenotypic aging measures’ derived from clinical biomarkers have been shown to be better predictors of whole-body aging and outcomes than actual age in representative population data [17–19].

Cd exposure has emerged as a significant contributor to the development of several diseases of aging, including diabetes [20], cardiovascular disease [21, 22], and osteoarthritis [19], as a result of the toxic effects of Cd on various metabolic organs [23]. Evidence from epidemiological studies and animal studies suggests that Cd exposure contributes to oxidative stress and stimulates the production of cytokines [24, 25], even though the exact mechanisms underlying these associations are still unknown. A recent study investigating the association between urinary metals and telomere length in the US population showed a negative association between U-Cd and telomere length [26].

To the best of our knowledge, no population-based investigation has studied the relationship between Cd exposure and phenotypic age. As a result, we conducted a cross-sectional study using data from the National Health and Nutrition Examination Survey (NHANES) to examine the link between Cd exposure and phenotypic age in a typical U.S. population.

## Methods

### Study population

**Fig. 1:**
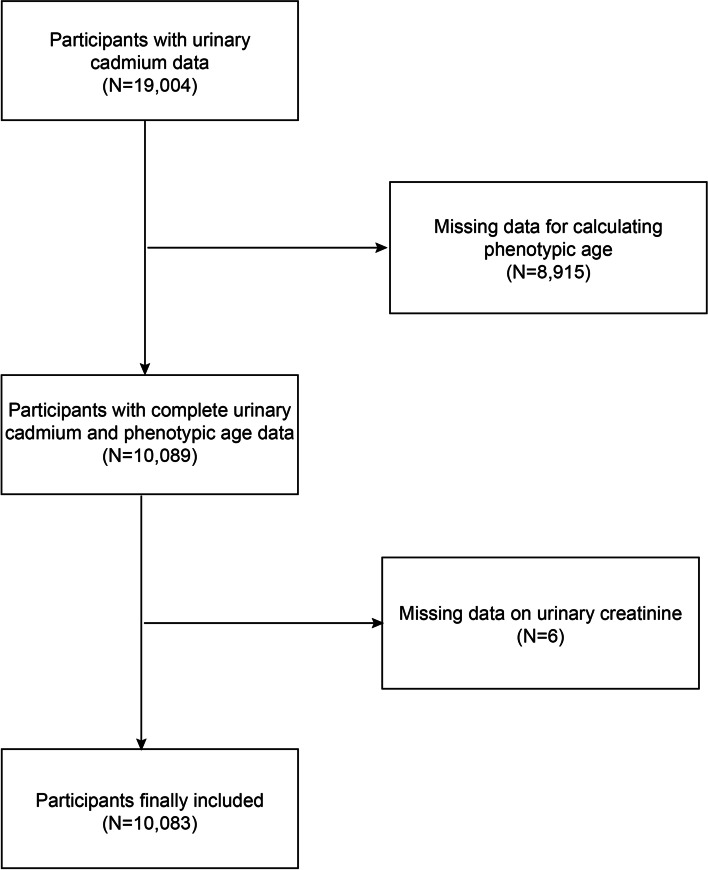
Flow chart of participants selection. NHANES, National Health and Nutrition Examination Survey

The National Center for Health Statistics (NCHS) conducts the well-known National Health and Nutrition Examination study (NHANES), a cross-sectional study that is nationally representative [27–29]. All research participants provided written agreement at the time of recruitment, and the NCHS Research Ethics Review Board approved the study’s methodology. Over 10 survey cycles in a period of twenty years (1999–2018), the survey was carried out. A total of 19,004 participants with U-Cd data were initially enrolled in the study, as were 8,915 participants without phenotypic age data and 6 participants with missing urinary creatine data. The study ultimately included 10,083 participants (Fig. 1).

### Cadmium exposure

In this study, urinary cadmium (U-Cd) levels were used as a Cd exposure assessment because it is considered a proxy for cumulative Cd exposure, reflecting the accumulation of Cd in the kidneys and other tissues [30]. U-Cd levels were measured by inductively coupled plasma mass spectrometry (ICP-MS), U-Cd concentration corrected by urinary creatinine [31, 32].

### Phenotypic age

The phenotypic age was determined using chronological age and nine biomarkers: albumin, creatinine, glucose, C-reactive protein, lymphocyte percentage, mean cell volume, erythrocyte distribution width, alkaline phosphatase, and white blood cell count [18]. Laboratory methods for measuring these biomarkers are as follows:

- Albumin: Measured using fluorescence immunoassay.
- Creatinine: Assessed by Jaffe kinetic method.
- Glucose: Measured using the glucose oxidase-peroxidase method.
- C-reactive protein: Quantified using a high-sensitivity enzyme-linked immunosorbent assay (ELISA).
- Percentage of lymphocytes: Calculated using flow cytometry.
- Mean cell volume: assessed using an automated hematology analyzer.
- Erythrocyte distribution width: Analyzed using an automated hematology analyzer.
- Alkaline phosphatase: Measured using colorimetric assay.
- Leukocyte count: Counted using an automated cell counter.

### Covariates

Covariates for this study were identified based on the literature on metal exposure and biological aging [33–35], including age, sex, education level, race, PIR (ratio of family income to poverty), BMI, smoking status (ever/never), sleep disorder, serum cotinine, cancer status, klotho, waist circumference, diabetes status, triglycerides and LDL-C (low-density lipoprotein cholesterol).

### Statistical analysis

All analyses were performed with R (version 4.2) and Empowerstats (version 5.0) [36, 37]. All statistical analyses were conducted weighted according to the NHANES guidelines. Missing covariate data were addressed using multiple imputation. To examine the demographic features of the individuals by U-Cd quartile, the chi-square test and t-test were utilized. Multivariate linear regression models were used to examine the linear associations between U-Cd and phenotypic age. The non-linear relationship between U-Cd and phenotypic age was investigated by smoothing curve fitting (penalized spline method) after logarithmic transformation of U-Cd [38]. Subgroup analyses and interaction tests were used to examine differences in the above correlations across gender, BMI, diabetes and age. Cd is a component of cigarette smoke [39], and Cd is also thought to be associated with aging [40]. Given that serum cotinine serves as an established biomarker for cigarette smoke exposure [41], to establish a foundation for mediation as described by Baron & Kenny [42], we first examined:

- The association between cotinine (independent variable) and U-Cd (mediator).
- The association between cotinine (independent variable) and phenotypic age (dependent variable).
- The results of these foundational analyses are presented in Table S1 of the supplementary material. Following the establishment of these associations, we conducted a mediation analysis to ascertain the extent to which Cd exposure mediates the relationship between serum cotinine and phenotypic age. The proportion of the effect mediated by Cd exposure was calculated using the formula (mediated effect/total effect) × 100%.

## Results

### Baseline characteristics

The mean (SD) age and phenotypic age of the 10,083 participants were 42.24 (21.55) years and 42.34 (21.93) years, with 49.76% of male participants. The mean U-Cd and serum cotinine was 0.33 (0.44) ng/g. The characteristics of the study population according to the quartiles of the U-Cd are depicted in Table 1. Participants in the higher urinary Cd quartiles were more likely to be older, female, non-Hispanic black, and smokers; have higher rates of diabetes and cancer; have higher serum cotinine levels, triglyceride levels, LDL-C levels, BMI, and waist circumference; and have lower educational levels and household income.

**Table 1:** Basic characteristics of participants by urinary cadmium quartile

Characteristics	Urinary Cadmium quartile (ng/g)				*P*-value
	Q1 (< 0.095) *N* = 2520	Q2 (0.095–0.198) *N* = 2504	Q3 (0.199–0.408) *N* = 2530	Q4 (> 0.409) *N* = 2529	
Age (years)	33.54 ± 17.21	40.74 ± 18.75	47.13 ± 17.77	54.08 ± 15.59	< 0.001
Phenotypic age (years)	33.61 ± 17.57	41.29 ± 18.98	47.24 ± 17.75	53.49 ± 15.64	< 0.001
Sex, (%)					< 0.001
Male	49.84	51.98	50.73	45.16	
Female	50.16	48.02	49.27	54.84	
Race/ethnicity, (%)					< 0.001
Non-Hispanic White	70.41	65.86	67.45	64.25	
Non-Hispanic Black	6.78	10.50	11.99	15.35	
Mexican American	10.04	11.24	8.79	7.41	
Other race/multiracial	12.77	12.39	11.77	12.98	
Education level, (%)					< 0.001
Less than high school	20.00	22.66	25.15	31.66	
High school	21.68	22.94	24.08	26.61	
More than high school	58.32	54.40	50.77	41.72	
Smoking, (%)					< 0.001
Ever	33.71	36.48	47.42	64.53	
Never	66.29	63.52	52.58	35.47	
Cancer, (%)					< 0.001
Yes	5.45	9.26	10.95	13.92	
No	94.55	90.74	89.05	86.08	
Diabetes, (%)					< 0.001
Yes	4.75	7.35	9.45	10.39	
No	94.21	91.39	87.57	87.38	
Borderline	1.04	1.26	2.98	2.23	
BMI (kg/m^2^)	26.78 ± 6.54	28.70 ± 6.67	29.29 ± 6.81	28.87 ± 6.86	< 0.001
Waist circumference (cm)	91.86 ± 16.85	97.50 ± 17.40	99.54 ± 16.76	99.61 ± 16.25	< 0.001
PIR	3.10 ± 1.62	3.04 ± 1.63	2.97 ± 1.63	2.74 ± 1.58	< 0.001
Triglycerides (mg/dL)	100.02 ± 76.60	112.73 ± 74.40	122.91 ± 89.35	134.96 ± 119.06	< 0.001
Klotho (pg/mL)	815.73 ± 313.40	831.34 ± 271.82	817.82 ± 277.08	813.23 ± 303.24	0.823
LDL-C (mg/dL)	99.63 ± 31.73	107.69 ± 35.76	115.25 ± 35.75	117.04 ± 35.26	< 0.001
Serum cotinine (ng/mL)	29.92 ± 95.40	36.25 ± 101.03	51.32 ± 122.73	98.92 ± 153.72	< 0.001

Mean ± SD for continuous variables: the P value was calculated by the weighted linear regression model

(%) for categorical variables: the P value was calculated by the weighted chi-square test

*Abbreviation*: *Q *Quartile, *PIR *Ratio of family income to poverty, *BMI *Body mass index, *LDL-C *Low-density lipoprotein cholesterol

### Associations between cadmium exposure and biological aging

Table 2 presents the results of multivariate linear regression models between U-Cd (ng/g) and phenotypic age (year). In the crude model, there was a positive correlation between U-Cd and phenotypic age, and the effect values were much higher than in the other models [13.97 (12.90, 15.05)], which mainly stemmed from the fact that the chronological age was not adjusted in Model 1. In the fully adjusted model, each 1 ng/g increase in U-Cd phenotypic age was associated with a 2.13-year increase in phenotypic age [2.13 (1.67, 2.58)]. When U-Cd was subsequently converted to quartiles and trend was tested, participants in the top quartile had phenotypic ages that were 1.6 years older than participants in the bottom quartile in the fully adjusted model [1.60 (1.00, 2.21)]. Additionally, all models showed a significant linear trend (P for trend < 0.01).

**Table 2:** The associations between urinary cadmium (ng/g) and phenotypic age (year)

Exposure	Model 1 [β (95% CI)]	Model 2 [β (95% CI)]	Model 3 [β (95% CI)]
Urinary cadmium (continuous)	13.97 (12.90, 15.05)	1.89 (1.52, 2.26)	2.13 (1.67, 2.58)
Urinary cadmium (quartile)			
Quartile 1	reference	reference	reference
Quartile 2	6.26 (4.86, 7.66)	0.32 (-0.17, 0.81)	0.04 (-0.57, 0.65)
Quartile 3	13.99 (12.63, 15.34)	0.79 (0.31, 1.28)	0.36 (-0.23, 0.95
Quartile 4	23.38 (22.03, 24.74)	2.04 (1.53, 2.56)	1.60 (1.00, 2.21)
P for trend	< 0.001	< 0.001	< 0.001

Model 1: no covariates were adjusted. Model 2: age, gender, and race were adjusted. Model 3: age, gender, race, BMI, smoking, alcohol drinking, diabetes, cancer, PIR, triglycerides, klotho, serum cotinine and LDL-C were adjusted

*Abbreviation*: *PIR *Ratio of family income to poverty, *BMI *Body mass index, *LDL-C *Low-density lipoprotein cholesterol

The subgroup analysis revealed that the link between U-Cd levels and phenotypic age was not consistent, despite the fact that a positive relationship occurred in all categories (Table 3). The results of the interaction test indicated that gender, age, and smoking modified the association between U-Cd levels and phenotypic age (P for interaction < 0.05). For participants who smoked, this positive linear association was strong and significant [2.27 (1.75, 2.78)], whereas in non-smokers, the association was not significant [0.97 (-0.02, 1.95)].

**Table 3:** Subgroup analysis of the association between urinary cadmium (ng/g) and phenotypic age (year)

Subgroup	Phenotypic age [β (95%CI)]	P for interaction
Sex		0.002
Male	2.81 (2.15, 3.48)	
Female	1.42 (0.82, 2.02)	
Age		0.016
< 60 years	1.77 (1.22, 2.33)	
≥ 60 years	2.93 (2.16, 3.71)	
Race/ethnicity		0.619
Non-Hispanic White	2.33 (1.77, 2.88)	
Non-Hispanic Black	1.85 (0.85, 2.85)	
Mexican American	1.38 (-0.57, 3.34)	
Other race/multiracial	1.60 (-0.00, 3.20)	
Education level, n (%)		0.579
Less than high school	2.55 (1.62, 3.48)	
High school	1.94 (1.22, 2.67)	
More than high school	2.31 (1.58, 3.03)	
BMI		0.185
< 24.9 kg/m^2^	2.44 (1.66, 3.22)	
25-29.9 kg/m^2^	1.67 (0.89, 2.44)	
≥ 30 kg/m^2^	2.61 (1.86, 3.36)	
Smoking, (%)		0.020
Ever	2.27 (1.75, 2.78)	
Never	0.97 (-0.02, 1.95)	
Cancer, (%)		0.278
Yes	1.51 (0.16, 2.85)	
No	2.29 (1.82, 2.77)	
Diabetes, (%)		0.088
Yes	1.55 (0.29, 2.80)	
No	2.30 (1.84, 2.76)	
Borderline	2.10 (1.08, 3.22)	

Age, gender, race, BMI, smoking, alcohol drinking, diabetes, cancer, PIR, triglycerides, klotho, serum cotinine and LDL-C were adjusted

*Abbreviation*: *PIR *Ratio of family income to poverty, *BMI *Body mass index, *LDL-C *Low-density lipoprotein cholesterol

**Fig. 2:**
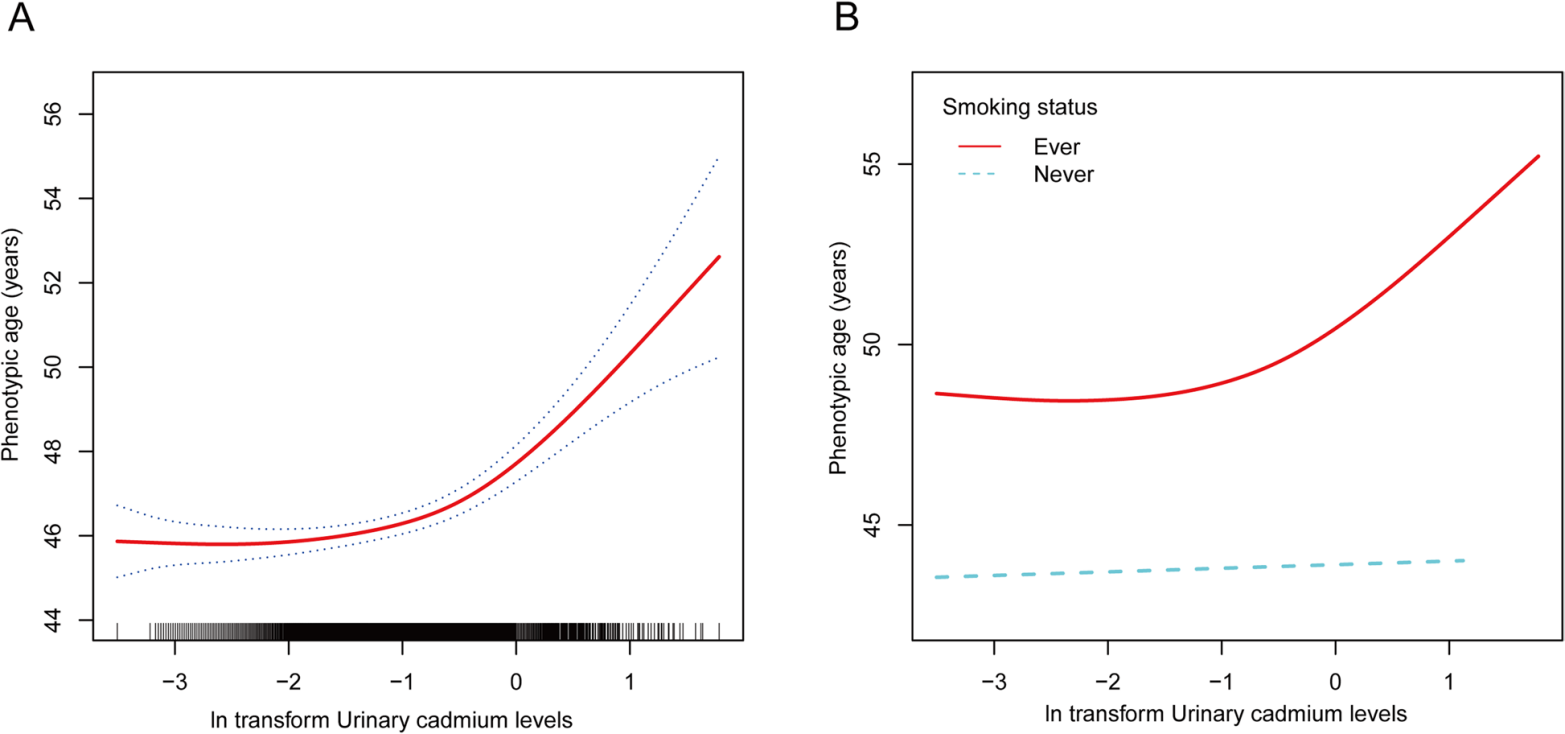
The nonlinear associations between urinary cadmium and phenotypic age. The solid red line represents the smooth curve fit between variables. Blue bands represent the 95% of confidence interval from the fit. **A** total participants; (**B**) Participants stratified by smoking

In addition, smoothed curve fitting further confirmed the nonlinear relationship between U-Cd and phenotypic age (Fig. 2). After stratification by smoking status, the nonlinear relationship between U-Cd and phenotypic age for participants who smoked showed a trend toward covariance with the nonlinear relationship for all participants, while non-smokers showed significant differences. Considering that smoking is one of the main sources of Cd exposure and that serum cotinine levels are a valid indicator of an individual’s smoking behavior and exposure, mediation analysis was further used to explore the role of U-Cd in mediating the relationship between serum cotinine and phenotypic age. U-Cd had a significant indirect effect (mediation effect) with a mediation ratio of 23.2% (Table 4).

**Table 4:** Urinary cadmium as a mediator in the associations between serum cotinine (ng/mL) and phenotypic age (year)

Mediation effect	Estimate	95% CI lower	95% CI upper	*P*-value
Total effect	0.012	0.008	0.013	< 0.001
Mediation effect	0.003	0.002	0.004	< 0.001
Direct effect	0.009	0.005	0.010	< 0.001
Proportion mediated	0.232	0.159	0.401	< 0.001

Model was adjusted for age, gender, race, BMI, alcohol drinking, diabetes, cancer, PIR, triglycerides, klotho, and LDL-C.

## Discussion

In past studies on biological aging, researchers have focused mainly on genetic factors and less on metal exposure. In this study, we provide two new findings based on a representative population in the United States. First, greater Cd levels were linked to biological aging, with each 1 ng/g rise in U-Cd related with a 2.13-year increase in phenotypic age. Also, Cd exposure had a mediating effect in the positive association of smoking on phenotypic age, with a 23.2% mediating proportion.

Phenotypic age is considered to be a valid indicator of whole-body aging [43], has been found to be associated with a range of health outcomes, including osteoarthritis [19], diabetes, and overall mortality [44]. These health outcomes may be due to changes in physiological and metabolic functions as a result of biological aging, including cellular senescence [45], decreased DNA repair capacity [46], and chronic inflammation [47]. In addition, phenotypic age may also be used as a risk assessment tool to identify individuals who are at high risk of developing health problems due to smoking or cadmium exposure [48]. For example, those with a higher phenotypic age than their actual age and with smoking or cadmium exposure may need to undergo more frequent health screenings or take more aggressive preventive measures. To our knowledge, this study is the first population-based study that investigated the association between Cd exposure and phenotypic age, and our results are consistent with many studies that have investigated other biological aging indicators [26, 49, 50]. Telomere shortening is an important mechanism of cellular senescence, and therefore telomere length is considered an important indicator of cellular senescence [51]. Patel et al. investigated the linear association of 461 variables, including environmental exposures, with telomere length, and their results showed that a total of eight of the 461 variables were associated with telomere shortening, including Cd exposure, C-reactive protein, and physical activity [49]. A cross-sectional study in China investigated the association of Cd and lead concentrations in the placenta with telomere length and demonstrated that telomere length was not connected with lead and was negatively associated with Cd concentration [50].

Furthermore, the results of the subgroup analysis of this study showed no association between U-Cd and phenotypic age in nonsmoking participants. In contrast, Demanelis et al. found an inverse association between Cd and biomarkers of aging in a non-smoking population [52]. These associations were significantly different from those in the smoking population, and these differences may stem from the increased levels of Cd exposure in humans due to smoking. The results of our mediation analysis showed that Cd exposure significantly mediated the positive association between smoking (serum cotinine) and phenotypic age, mediating a proportion of 23.2%. Cigarette smoke is thought to be an important factor in accelerating the aging process [53], and the association between nicotine metabolites and phenotypic age may be due in part to the presence of Cd in tobacco smoke. Even though our study was cross-sectional and we were unable to establish a causal link, this interpretation is supported by the findings of the mediation analysis and a number of experimental studies [54, 55]. In addition, the mediating effect of Cd exposure between smoking and telomere length was also demonstrated in a cross-sectional study by Zota et al. using mediation analysis, but their measure of smoking utilized questionnaire variables, years of smoking (30 years, 30–59 years, > 60 years) [56], whereas we investigated the mediating effect of smoking between Cd exposure and phenotypic age from a different perspective using the nicotine metabolite serum cotinine.

There are many biological aging mechanisms that have been linked to Cd exposure. It is believed that oxidative stress is the main cause of telomere shortening. High levels of guanine in telomeres, which are extremely vulnerable to reactive oxygen species, cause the production of 8-oxo-7,8-dihydrodeoxyguanosine, which can cause DNA strand breaks and telomere wear [57–59]. In addition, Cd exposure is associated with higher levels of inflammatory markers [60, 61], and inflammation may further induce oxidative stress to accelerate cellular senescence [62]. Finally, Cd has been shown to have the ability to interfere with the DNA repair system and can affect the stability of excision and mismatch repair systems [63].

Our research has several limitations. First, due to the cross-sectional study’s design, we were unable to establish a causal association between Cd exposure and biological aging. Furthermore, the variables related with biological aging are too complicated for us to account for all potential confounding factors, such as medication usage and food recall, which may significantly influence the results. Despite these shortcomings, our study provides a number of advantages. The current study is the first to investigate the association between Cd exposure levels and whole-body aging. In addition, a large representative sample size was included in this study, which allowed us to stratify the analysis across multiple variables and reduce the error in the results of the subgroup analysis.

## Conclusion

Cd exposure is positively associated with whole-body aging (phenotypic age). In addition, U-Cd mediated a positive association between smoking and whole-body aging. These results suggest that phenotypic age may be used as a risk assessment tool to identify individuals who are at high risk of developing health problems due to smoking or cadmium exposure.

### Supplementary Information

**Additional file 1: Table S1. **The associations between cotinine (ng/mL) with urinary cadmium (ng/g) and phenotypic age (year).

## Data Availability

The survey data are publicly available on the internet for data users and researchers throughout the world ( www.cdc.gov/nchs/nhanes/ ).

## Abbreviations

U-Cd: Urinary cadmium
NHANES: National Health and Nutrition Examination Survey
NCHS: National Center for Health Statistics
ICP-MS: Inductively coupled plasma mass spectrometry
LDL-C: Low-density lipoprotein cholesterol
BMI: Body mass index
PIR: Ratio of family income to poverty
