# Supplementary material for: Associations between cadmium exposure and whole-body aging: mediation analysis in the NHANES
Source: BMC Public Health. 2023 Aug 31;23:1675. doi: 10.1186/s12889-023-16643-2 (PMC10469832; doi:10.1186/s12889-023-16643-2)
Supplement: Supplementary file 1 — Additional file 1: Table S1. The associations between cotinine (ng/mL) with urinary cadmium (ng/g) and phenotypic age (year). [file 12889_2023_16643_MOESM1_ESM.docx]

**Table S1:** The associations between cotinine (ng/mL) with urinary cadmium (ng/g) and phenotypic age (year).

| Cotinine | **Model 1 [β (95% CI)]** | **Model 2 [β (95% CI)]** | **Model 3 [β (95% CI)]** |
| --- | --- | --- | --- |
| Urinary cadmium |  |  |  |
| Tertile 1 | reference | reference | reference |
| Tertile 2 | 0.04 (0.02, 0.06) | 0.06 (0.04, 0.08) | 0.05 (0.03, 0.08) |
| Tertile 3 | 0.17 (0.15, 0.19) | 0.22 (0.20, 0.24) | 0.23 (0.20, 0.25) |
| Phenotypic age |  |  |  |
| Tertile 1 | reference | reference | reference |
| Tertile 2 | -2.09 (-3.41, -0.76) | 0.45 (0.03, 0.87) | 0.27 (-0.26, 0.79) |
| Tertile 3 | -4.65 (-5.97, -3.33) | 2.34 (1.91, 2.77) | 1.95 (1.39, 2.51) |

Model 1: no covariates were adjusted. Model 2: age, gender, and race were adjusted. Model 3: age, gender, race, BMI, smoking, alcohol drinking, diabetes, cancer, PIR, triglycerides, klotho, and LDL-C were adjusted. Abbreviation: PIR, Ratio of family income to poverty; BMI, body mass index; LDL-C, low-density lipoprotein cholesterol.
